# Effects of canagliflozin on cardiac remodeling and hemodynamic parameters in patients with type 2 diabetes mellitus

**DOI:** 10.1038/s41598-023-48716-y

**Published:** 2023-12-03

**Authors:** Hsiao-Huai Kuo, Yau-Huei Lai, Po-Lin Lin, Hsin-Hao Chen, Chung-Lieh Hung, Lawrence Yu-Min Liu, Chih-Kuang Yeh

**Affiliations:** 1 Department of Biomedical Engineering and Environmental Sciences, National Tsing Hua University, Hsinchu, Taiwan; 2 Department of Pharmacy, Hsinchu Municipal MacKay Children’s Hospital, Hsinchu, Taiwan; 3 Department of Pharmacy, Hsinchu MacKay Memorial Hospital, Hsinchu, Taiwan; 4 Department of Nursing, Hsin Sheng Junior College of Medical Care and Management, Taoyuan, Taiwan; 5 Department of Medicine, MacKay Medical College, New Taipei City, Taiwan; 6 Division of Cardiology, Department of Medicine, Hsinchu MacKay Memorial Hospital, Hsinchu, Taiwan; 7 MacKay Junior College of Medicine, Nursing, and Management, Taipei, Taiwan; 8 Yuanpei University of Medical Technology, Hsinchu, Taiwan; 9 Department of Family Medicine, Hsinchu MacKay Memorial Hospital, Hsinchu, Taiwan; 10 Division of Cardiology, Department of Internal Medicine, MacKay Memorial Hospital, Taipei, Taiwan

**Keywords:** Cardiology, Endocrinology

## Abstract

Sodium-glucose cotransporter type 2 (SGLT2) inhibitors have demonstrated to reduce cardiovascular risk in patients with type 2 diabetes mellitus (T2DM) in large trials independent of glycemic control. The mechanisms of this cardioprotective property remain uncertain. Evidence suggests positive hemodynamic changes and favorable cardiac remodeling contributing to the clinical outcomes but results were conflicting. We aim to investigate the potential impact on hemodynamic parameters, cardiac structure and functions. This prospective observational study included T2DM patients receiving canagliflozin 100 mg per day in addition to their antidiabetic treatment. We analyzed hemodynamic parameters assessed by echocardiographic measurements and impedance cardiography (ICG) to evaluate systolic and diastolic functions from baseline to 24 weeks after treatment. A total of 47 patients (25 males and 22 females) averaging 64.6 ± 10.9 years had a significant reduction in HbA1c, body weight, and systolic blood pressure. Hematocrit increased significantly, while NT-proBNP remained unchanged. E/e′, left atrium (LA) volume, and LA stiffness were reduced, while left ventricle (LV) global longitudinal strain (GLS) and LA strain rates increased at 24 weeks by conventional and speckle tracking echocardiography. LV mass and ejection fraction showed no differences. ICG suggested significant improvement in hemodynamic parameters with increased stroke volume index and cardiac output index and decreased systemic vascular resistance index at 12 and 24 weeks. Canagliflozin improved hemodynamic parameters and had a favorable impact on LA and LV reverse remodeling. These changes may explain the beneficial effect on cardiovascular outcomes in large clinical trials.

## Introduction

Sodium-glucose cotransporter type 2 (SGLT2) inhibitors are a novel class of oral diabetes drugs that block the reabsorption of glucose via SGLT2 in the proximal tubules of the kidneys, allowing glucose to be excreted in the urine, and achieve the therapeutic effect of lowering blood sugar^1^. Additionally, they reduce body weight, blood pressure, and uric acid level, independent of their hypoglycemic effects. Several SGLT2 inhibitors, such as empagliflozin, dapagliflozin, and canagliflozin, have been found to have cardiovascular benefits in large placebo-controlled clinical trials and are the first oral diabetes drugs that can reduce cardiovascular risk^2, 3^. In contrast, other hypoglycemic drugs like dipeptidyl peptidase-4 inhibitors (DDP4i) and thiazolidinediones are ineffective in cardiovascular risk reduction and are even associated with increased heart failure in patients with type 2 diabetes mellitus (T2DM).

The most significant and consistent cardiovascular outcome from SGLT2 inhibitors is the reduction of hospitalization for heart failure (HHF). Recent meta-analyses of cardiovascular outcome trials show a 32–33% reduction of HHF without heterogeneity, and this effect extends to patients without type 2 diabetes^4, 5^. EMPEROR-Preserved further demonstrates the benefits in patients with heart failure and a preserved ejection fraction (HFpEF) with a 29% lower risk of HHF with empagliflozin^6^. Moreover, SGLT2 inhibitors have impressive renal outcomes with improvement in albuminuria, declining kidney function, and renal death independent of glycemic control and the presence of diabetes^7, 8^.

Despite the cardiovascular benefits of SGLT2 inhibitors, the underlying mechanisms of action remain unclear and are to be elucidated. Several hypotheses have been postulated to explain these cardiorenal benefits, including diuresis effect, hemodynamic improvement, or augmented cardiac metabolism^9, 10^.

Impedance cardiography (ICG) is a convenient, non-invasive method to approximate valuable hemodynamic parameters and is often used in surgical and intensive care units to guide therapy^11^. It utilizes skin sensors placed on the neck and chest to measure changes in electrical conductivity within the thorax to calculate cardiac output, stroke volume, systemic vascular resistance (SVR), thoracic fluid status, and other contractility indexes^12^. Previous studies have demonstrated that ICG improved and predicted clinical outcomes in patients with acute and chronic heart failure^13, 14^. Several parameters measured by ICG also predicted mortality in the general population, even after adjustment for multiple confounders and blood pressure^15^.

Our observational study aims to assess the effect of canagliflozin on hemodynamic parameters measured by ICG and left ventricular functions evaluated by echocardiography in patients with T2DM. The results of this study will provide valuable insights into the mechanisms of action of SGLT2 inhibitors and their effects on cardiovascular and renal outcomes in patients with T2DM.

## Methods

### Study design

This prospective, single-center observational study was conducted at Hsinchu MacKay Memorial Hospital, a regional hospital in Northern Taiwan, to investigate the effect of canagliflozin on patients with T2DM. The study was approved by the Institutional Review Board of MacKay Memorial Hospital (Registration Number: 18MMHIS162e) and followed the principles of the Declaration of Helsinki. Each study participant provided informed consent. The enrolled patients with T2DM who were SGLT2 inhibitor-naive received canagliflozin 100 mg once daily in addition to previously prescribed antidiabetic agents. Physical examinations and hemodynamic monitoring were performed at baseline and in the 4th, 12th, and 24th weeks after administration of canagliflozin. Body weight and fat mass were measured using a commercially available scale with a bioelectrical impedance-based (BIA) body composition monitor (Tanita-305 Body-Fat Analyzer, Tanita Crop, Tokyo, Japan). Blood tests were conducted at baseline and in the 12th and 24th weeks, except for hs-CRP, aldosterone, and renin, which were measured at baseline and in the 24th week only. Echocardiography was performed at baseline and in the 24th week.

### Participants

The target patient population was T2DM patients who had not taken any SGLT2 inhibitor prior to the study. These patients were recruited from outpatients at Hsinchu Mackay Memorial Hospital, Taiwan, and received standard care for T2DM during the study period. Other inclusion criteria were: (1) age ≥ 20 years old, (2) eGFR ≥ 30 mL/minute/1.73 m^2^, and (3) patients taking metformin alone or taking metformin and other antidiabetic drugs. The key exclusion criteria included: (1) patients with type 1 diabetes, (2) pregnant or breastfeeding patients, (3) New York Heart Association (NYHA) Class III and IV, (4) acute or chronic inflammatory disease, (5) patients who are allergic to canagliflozin and its excipients, and (6) severe obesity (BMI ≥ 40 kg/m^2^).

### Echocardiography

Echocardiography was performed by a single trained sonographer blinded to clinical information using a commercially available ultrasound system (GE Vivid 7, GE Medical System, Vingmed, Norway). Standardized echocardiographic measurements were obtained according to the American Society of Echocardiography (ASE) recommendations. Left atrium (LA) and left ventricle (LV) volumes were obtained via the modified biplane Simpson method from the apical two- and four-chamber views. Diastolic function was assessed from early mitral inflow velocity (E), late mitral inflow velocity (A), deceleration time (DT), and tissue Doppler imaging (TDI)-derived mitral annular early diastolic velocity (E′) from LV septal and lateral segments. The mean values of septal and lateral E′ were reported in our tables.

LV and LA strains were assessed by speckle tracking analysis. Baseline 2D images were analyzed offline by manually tracing the endocardial border using proprietary software (version 10.8, EchoPAC, GE Vingmed Ultrasound, Norway). Based on automated speckle-tracking algorithms, LV global longitudinal strain (LV GLS) curves were obtained from the three standard apical views (long-axis, 4-chamber, and 2-chamber). Peak atrial longitudinal strain (PALS) and strain rate (SR) curves (systolic, early, and late diastolic LA strain rate [LA SRs, SRe, and SRa, respectively]) were generated for each atrial segment from apical 2- and 4-chamber views. The ratio of E/e′ to peak LA strain was used to estimate LA stiffness. Minor manual adjustment was used as necessary to ensure the best tracking quality. The absolute values of longitudinal strain (instead of negative values) were reported in this manuscript, and the corresponding tables and figures to avoid confusion regarding the directionality of the strain changes.

### Hemodynamic monitoring

The non-invasive cardiac hemodynamic measuring instrument, AESCULON (OSYPKA Medical, USA), was used to provide the hemodynamic parameters. The device sends a low-amplitude, high-frequency electrical current through the thorax, and two sensors attached to the neck and another two to the left side of the thorax measure the changes in conductivity created by the circulatory system. The hemodynamic parameters were available after three minutes, and the report was printed for analysis. Table 3 contains the detailed variables evaluated in this study.

### Statistical analysis

Data were expressed as mean ± stand deviation for continuous variables and numbers (%) for categorical variables. The comparison of each parameter before and after treatment with canagliflozin was calculated by paired sample t-test. All statistics were two-sided and statistically significant at *p* < 0.05.

Statistical analyses were performed using IBM SPSS Statistics for Windows, version 24.0 (IBMCorp., Armonk, NY, USA).

### Ethics approval and consent to participate

The study was approved by the Institutional Review Board of MacKay Memorial Hospital (Registration Number: 18MMHIS162e).

## Results

### Baseline characteristics

From November 2019 to February 2021, 52 T2DM patients were included in the study, and five patients did not complete the study. Three stopped the medication because of adverse drug effects (1 due to dysuria, two due to palpitation), one could not have the examinations due to Covid travel restrictions, and one withdrew consent. Table 1 shows the clinical characteristics of the 47 patients analyzed at baseline.

**Table 1:** Baseline characteristics of patients.

Baseline characteristics of patients (n = 47)	
Age, mean (SD), year	64.6 (10.9)
Male, n(%)	25 (53)
Weight (kg)	74.1 (13.5)
Body mass index, mean (SD), kg/m^2^	28.2 (4.2)
Fat content, mean (SD), kg	23.3 (10.0)
Systolic blood pressure, mean (SD), mmHg	137.2 (16.0)
Diastolic blood pressure, mean (SD), mmHg	79.3 (12.1)
Heart rate, mean (SD), beats/min	75.5 (12.1)
Glycemic status	
HbA1c, mean (SD)	7.5 (1.5)
Fasting blood glucose, mean (SD), mg/dL	143 (49)
Duration of diabetes, mean (IQR), year	6.3 (7.8)
Hypertension, n (%)	38 (80.9)
Hyperlipidemia, n (%)	41 (87.2)
Chronic kidney disease	38 (80.9)
History of cardiovascular diseases, n (%)	34 (72.3)
Coronary artery disease	27 (57.4)
Congestive heart failure	15 (31.9)
Cerebrovascular disease	3 (6%)
Antidiabetic drugs, n (%)	
Metformin, n (%)	37 (78.7)
Sulfonylureas, n (%)	23 (48.9)
DPP-4 inhibitors, n (%)	4 (8.5)
α-glucosidase inhibitors, n (%)	2 (4.3)
Thiazolidinediones, n (%)	0
Glinides, n (%)	0
Insulin, n (%)	6 (12.8)
GLP-1 receptor agonists, n(%)	1 (2.1)
Antihypertensive agents, n (%)	
RAAS inhibitors, n (%)	36 (76.6)
Calcium channel blockers, n (%)	29 (61.7)
Beta blockers, n (%)	30 (63.8)
Alpha blockers, n (%)	4 (8.5)
Diuretics, n (%)	10 (21.3)
Statins, n (%)	38 (80.9)
Fibrates, n (%)	6 (12.8)

The data are expressed as number of patients (percentage). *DPP-4* dipeptidyl peptidase 4, *GLP-1RA* glucagon like peptide-1 receptor agonist. *RAAS* renin–angiotensin–aldosterone system.

The mean age of the participants was 64.6 ± 10.9 years. There were 25 (53%) males and 22 (47%) females. The mean body mass index (BMI) and fat content were 28.2 kg/m^2^ and 23.3 kg, respectively, indicating that the patients were overweight on average and some had a high body fat percentage. The majority of patients had a history of cardiovascular disease (95.7%), and most were taking antihypertensive medications and statins. The patient’s medications, including antihypertensives, diuretics, and antidiabetic agents, remained similar during the study period.

The mean weight changed from 74.1 ± 13.4 to 72.6 ± 13.8 kg, and the mean body mass index (BMI) 28.2 ± 4.3 to 27.6 ± 4.3 kg/m^2^, both statistically significant. However, the fat mass and ratio measured by BIA showed no difference 6 months after the administration of canagliflozin. Both fasting blood glucose level (141.2 ± 45.3–135.7 ± 57.9 mg/dL, *p* < 0.001) and HbA1c (7.5 ± 1.4–7.0 ± 1.0%, *p* = 0.008) showed a considerable drop. The mean hemoglobin (13.5 ± 1.4–14.1 ± 1.4 g/dL, *p* < 0.001) and hematocrit (40.1 ± 3.6–42.5 ± 4.1%, *p* < 0.001) increased at 6 months. The above changes were observed and statistically significant at 12 weeks and continued until the end of the study. There was a trend for reduction of microalbuminuria/creatine ratio, albeit statistically insignificant. No difference was seen in lipid profiles, renal functions, electrolytes, uric acid, levels of NT-proBNP, hs-CRP, renin, and aldosterone. Clinical changes are shown in Table 2.

**Table 2:** Comparison of variables between baseline, 12th and 24th weeks after administration of canagliflozin.

	Baseline mean (SD)	12-Week mean (SD)	*p*-value	24-Week Mean (SD)	*p*-value
Weight (kg)	74.05 ± 13.40	72.85 ± 13.70	< 0.001	72.59 ± 13.80	< 0.001
Body mass index (kg/m^2^)	28.22 ± 4.30	27.75 ± 4.30	< 0.001	27.56 ± 4.30	< 0.001
Fat ratio (%)	30.82 ± 11.50	30.72 ± 10.30	0.895	30.15 ± 11.00	0.781
HbA1c (%)	7.50 ± 1.40	7.00 ± 0.80	0.01	7.00 ± 1.00	0.008
Fasting glucose (mg/dL)	141.2 ± 45.3	126.8 ± 30.4	0.004	135.7 ± 57.9	< 0.001
GOT	31.2 ± 17.1	27.2 ± 16.6	0.087	27.1 ± 17.6	0.099
GPT	34.5 ± 20.6	27.4 ± 12.8	0.008	28.4 ± 14.8	0.034
BUN (mg/dL)	17.4 ± 5.5	19.4 ± 6.0	0.002	20.3 ± 7.2	0.014
Creatinine (mg/dL)	1.0 ± 0.3	1.0 ± 0.3	0.292	1.0 ± 0.3	0.49
Estimated GFR (mL/min/1.73m^2^)	68.7 ± 17.6	68.4 ± 21.4	0.844	67.7 ± 19.4	0.983
Sodium	137.7 ± 2.4	138.1 ± 1.8	0.146	137.5 ± 1.9	0.642
Potassium	4.3 ± 0.4	4.3 ± 0.5	0.747	4.4 ± 0.5	0.150
Uric acid	5.9 ± 1.3	5.8 ± 1.6	0.723	6.1 ± 1.7	0.759
Triglyceride (mg/dL)	145.7 ± 94.4	148.1 ± 115.5	0.869	160.3 ± 112.4	0.401
Microalb/Crea Ratio	325.2 ± 786.7	238.6 ± 510.8	0.116	245.9 ± 751.8	0.176
LDL (mg/dL)	78.7 ± 23.1	76.6 ± 22.9	0.584	74.3 ± 25.4	0.371
HDL (mg/dL)	44.7 ± 13.3	43.6 ± 12.5	0.241	42.7 ± 10.6	0.295
Hematocrit (%)	40.1 ± 3.6	41.5 ± 4.3	< 0.001	42.5 ± 4.1	< 0.001
NT-proBNP	113.4 ± 121.6	131.5 ± 166	0.238	124.0 ± 134.4	0.688
Renin	3.2 ± 5.2	na	na	5.8 ± 10.0	0.096
Aldosterone	105.4 ± 89.7	na	na	111.5 ± 71.8	0.605

Data is mean ± SD for normally distributed data and median and interquartile range for non-normally distributed data.

*GFR* glomerular filtration rate, *LDL* low density lipoprotein, *HDL* high density lipoprotein, *NT-proBNP* N terminal pro B type natriuretic peptide.

### Echocardiography

There were no significant differences in the LV mass, LV volume, LV ejection fraction, and cardiac output derived from echocardiography 24 weeks after the administration of canagliflozin (Table 3). Nevertheless, the LV diastolic function assessed by E/e′ ratio improved significantly (11.3 ± 3.2–9.1 ± 2.9, *p* < 0.001). LV global longitudinal strain (GLS), LV systolic and early strain rate (LVSRs and LVSRe) also significantly increased from 16.6 ± 2.8 to 18.0 ± 3.4 (*p* < 0.001), 1.08 ± 0.31 to 1.15 ± 0.35 (*p* = 0.013), and 0.90 ± 0.25 to 1.09 ± 0.31 (*p* < 0.001), respectively. Moreover, LA volume and LA stiffness decreased while LA systolic function assessed by PALS and LA strain rates increased with canagliflozin treatment at 24 weeks. Data are shown in Table 3.

**Table 3:** Changes of echocardiographic parameters from baseline to Week 24.

	Baseline Mean (SD)	24-Week mean (SD)	*p* value
LV mass (g)	182.1 ± 51.8	187.1 ± 51.0	0.520
LV EDV (mL)	105.7 ± 30.7	108.2 ± 33.1	0.649
LV ESV (mL)	41.0 ± 17.8	41.0 ± 17.2	0.982
Left ventricular Ejection Fraction (%)	61.8 ± 8.3	62.8 ± 7.5	0.308
Cardiac output (mL)	4.9 ± 1.6	4.9 ± 1.9	0.971
Stroke volume index	36.6 ± 10.2	38.4 ± 10.7	0.345
LV IVRT (ms)	94.2 ± 11.3	96.5 ± 23.0	0.525
DT (ms)	220.9 ± 48.3	226.1 ± 46.1	0.492
E (m/s)	0.8 ± 0.2	0.7 ± 0.2	0.262
A (m/s)	0.9 ± 0.2	0.9 ± 0.2	0.622
E/e' ratio	11.3 ± 3.2	9.1 ± 2.9	< 0.001
LV GLS (%)	16.6 ± 2.8	18.0 ± 3.4	< 0.001
LA Volume (mL)	51.8 ± 16.5	46.6 ± 15.3	< 0.001
PALS (%)	23.6 ± 5.8	28.2 ± 7.4	< 0.001
LA SRs	1.3 ± 0.4	1.4 ± 0.5	0.006
LA SRe	0.9 ± 0.3	1.3 ± 0.4	0.000
LA SRa	1.3 ± 0.5	1.3 ± 0.6	0.611
LA stiffness	0.5 ± 0.2	0.4 ± 0.2	< 0.001

*EDV* end-diastolic volume, *ESV* end-systolic volume, *IVRT* isovolumic relaxation time, *DT* deceleration time, *GLS* global longitudinal strain, *PALS* peak atrial longitudinal strain, *LA SRs* systolic LA strain rate, *LA SRe* early LA strain rate, *LA SRa* late LA strain rate.

### Hemodynamic monitoring

The hemodynamic parameters by ICG at baseline, 4 weeks, 12 weeks, and 24 weeks of canagliflozin are summarized in Table 4. The mean systolic blood pressure decreased significantly at 24 weeks (136.4 ± 15.4–130.7 ± 14.8 mmHg, *p* = 0.019). The average heart rate also dropped at 24 weeks from 75.0 ± 11.3 bpm to 71.5 ± 12.4 bpm (*p* = 0.003).

**Table 4:** Comparison of hemodynamic variables between baseline and 12th and 24th weeks after administration canagliflozin.

Hemodynamic variables	Baseline mean (SD)	12-Week mean (SD)	*p*-value	24-Week mean (SD)	*p*-value
Heart rate (beats/min)	75.0 ± 11.3	72.5 ± 13.2	0.043	71.5 ± 12.4	0.003
Systolic BP	136.4 ± 15.4	135.0 ± 19.0	0.641	130.7 ± 14.8	0.019
Diastolic BP	78.9 ± 11.3	77.5 ± 11.3	0.304	78.1 ± 12.2	0.469
Mean BP	98.1 ± 11.4	96.7 ± 12.4	0.421	95.7 ± 11.1	0.109
Cardiac index	2.5 ± 0.6	2.6 ± 0.6	0.022	2.9 ± 0.7	< 0.001
Cardiac output (L/min)	4.4 ± 1.0	4.7 ± 1.2	0.021	5.1 ± 1.5	< 0.001
Stroke index[BSA]	33.4 ± 6.4	36.6 ± 6.2	< 0.001	40.3 ± 7.7	< 0.001
Stroke volume (mL)	59.6 ± 12.0	64.9 ± 11.3	0.001	71.8 ± 16.5	< 0.001
Systemic vascular resistance index[BSA]	3301.2 ± 788.5	3042.3 ± 873.5	0.014	2796.7 ± 797.3	< 0.001
Thoracic fluid content	24.6 ± 5.3	27.5 ± 6.1	0.009	32.7 ± 7.1	< 0.001
Index of contractility (ICONTM)	37.1 ± 12.7	44.6 ± 12.8	< 0.001	49.2 ± 13.4	< 0.001
Systolic time ratio	0.4 ± 0.1	0.4 ± 0.1	0.268	0.4 ± 0.1	0.705
Left ventricular ejection time (ms)	295.8 ± 21.8	296.3 ± 25.5	0.842	296.4 ± 23.8	0.288
Pre ejection time (ms)	108.5 ± 23.5	103.4 ± 21.0	0.161	107.1 ± 20.7	0.884

systolic time ratio = pre-ejection time/left ventricular ejection time.

ICG parameters of blood flow and vascular system, including cardiac output, cardiac index, stroke volume, stroke index, and systemic vascular resistance index, showed significant improvement at 12 and 24 weeks after starting canagliflozin. The index of contractility (ICON™) increased significantly, but the other parameters of contractility, including left ventricular ejection time, pre-ejection time, and systolic time ratio, did not change. Unexpectedly, thoracic fluid content (TFC), which reflects the extravascular and intravascular fluid, increased from 24.6 ± 5.3 to 32.7 ± 7.1 k/Ω (*p* < 0.001).

## Discussion

In this prospective study, canagliflozin significantly improved the hemodynamic parameters of blood flow, including stroke volume, stroke index, cardiac output, and cardiac index, as well as systemic vascular resistance by ICG. Furthermore, we observed the beneficial effects of canagliflozin on LV diastolic function and subtle improvement in LA and LV systolic functions evaluated by speckle tracking.

The mechanisms underpinning the overwhelming effect of cardiorenal protection by SGLT2 inhibitors are not yet fully understood. It is postulated that SGLT2 inhibitors may augment erythropoiesis and suppress sympathetic hyperactivity^16–18^, which are supported by the elevation of hematocrit and reduction of the resting HR and SVRI in this study. Elevated levels of erythropoietin (EPO) increased erythropoiesis and hematocrit and helped reduce the risk of heart failure and ischemic heart disease^19, 20^. The reduction of afterload by lowering blood pressure and decreasing vascular resistance likely contributed to the observed overall increase in hemodynamic parameters of blood flow, as well as the improvements in subclinical systolic functions. Studies have demonstrated that SGLT2 inhibitors can directly improver vascular function by inducing vasorelaxation and reducing endothelial cell dysfunction associated with atherogenesis^21, 22^. Moreover, the “fuel hypothesis” suggests that SLGT2 inhibitors increase ketone bodies, thereby optimizing cardiac energy metabolism and cardiac function may ameliorate the outcomes^23, 24^.

Elevated E/e′ is a valuable marker for diagnosing heart failure with preserved ejection fraction and predicting prognosis^25^. The decrease in E/e′, indicative of decreased LV filling pressure and diastolic function improvement, is a consistent finding observed in this and other SGLT2 inhibitors’ studies^26–28^. In the IDDIA trial, E/e′ only decreased significantly during exercise in T2DM patients with dapagliflozin^29^. The other echocardiographic parameters for diastolic function, including LV mass, LA volume, and E/A ratio, are less sensitive. MRI imaging also did not detect an apparent change in the LA or LV geometry^30, 31^. Nevertheless, our data from speckle tracking provided additional evidence suggesting beneficial effects on cardiac remodeling and function. LV GLS increased significantly while LVEF did not change, suggesting early and subclinical LV systolic improvement, which was similarly found in other studies^32, 33^. The administration of canagliflozin was also associated with significant improvement in LA strain indices and LA stiffness.

To our knowledge, few prior studies reported a comprehensive analysis of LA deformation with the administration of SGLT2 inhibitors. Decreased LA strain and increased LA stiffness have been strongly correlated with atrial fibrillation and adverse clinical outcomes in patients with heart failure with reduced EF (HFrEF) or HFpEF^34, 35^. However, our findings contradicted previous studies that found empagliflozin and dapagliflozin had no effect on any hemodynamic parameters evaluated by ICG in T2DM patients at 3 months and 12 weeks, respectively^28, 36^. Although all studies enrolled T2DM patients without overt heart failure, there were differences in baseline characteristics. Patients in our study were predominately female, had smaller BMIs, better diabetic control, greater reductions of SBP, and received less diuretic therapy. Additionally, the number of patients enrolled in these studies was limited.

Another possible explanation is that canagliflozin is more selective towards SGLT1 receptors, which are expressed abundantly in the distal proximal tubules of kidneys, the small intestine, and the cardiomyocytes. Suppression of SGLT1 can facilitate glucose uptake, decrease oxidative stress, reduce myocardial fibrosis and ventricular hypertrophy, and improve cardiac function^37, 38^. A previous study has shown oxidative stress was strongly associated with impaired myocardial performance and decreased GLS^39^. Dual SGLT1/2 inhibitor, sotagliflozin, has also been shown to reduce the overall risk of cardiovascular death, hospitalization for HF and emergent HF visit^40, 41^. Thus, the additional SGLT1 inhibition may potentiate the hemodynamic effect of canagliflozin.

Large clinical trials have recognized the effect of SGLT2 inhibitors in reducing the risk of hospitalization for heart failure, regardless of the presence of diabetes or glycemic control. Furthermore, SGLT2 inhibitors have been found to be effective in non-diabetic patients with HFrEF or HFpEF, who previously had no effective therapy^6, 42–44^. Our data suggest that positive cardiac remodeling, improved LV systolic and diastolic functions, and reduced systemic vascular resistance contribute to the broad therapeutic efficacy of SGLT2 inhibitors. Although it is postulated that SGLT2 inhibitors exert a diuretic effect that removes interstitial fluid to reduce the risk of HF, this effect is transient and modest and cannot account for the long-term outcome in clinical trials. In our study cohort, baseline measurements revealed normal level of Nt-proBNP and relatively low TFC. Interestingly, the level of Nt-proBNP did not decrease, and TFC even exhibited an elevation, despite positive hemodynamic changes observed after 6 months of canagliflozin treatment. These findings suggest that other mechanisms may be responsible for the therapeutic effect of SGLT2 inhibitors. The observed increase in TFC may be attributed to the counteracting mechanism of fluid regulation following the inhibition of SGLT2, as evident by the trending elevation in plasma renin activity^45^.

This study has several limitations. Firstly, the non-randomized, uncontrolled design affects the validity of our findings. Secondly, the small sample size and short follow-up duration may limit the generalizability of our results. A large randomized controlled trial is needed to confirm the positive hemodynamic effects of canagliflozin. The small percentage of patients with heart failure also made it difficult to detect the subclinical difference in some endpoints by conventional echocardiography over the 6-month period. Lastly, ICG utilizes the changes in electrical conductivity of the aortic arch blood flow detected by the skin sensors to estimate stroke volume and cardiac output^46^. A recent meta-analysis has indicated limitations in the inability of electrical cardiometry to measure absolute cardiac output values, and it may be applicable as a trend monitor if performed precisely^47^. Factors affecting the tissue and device conductivity, time and postural variability, and different algorithms may influence the calculated values. Nevertheless, our clinical and echocardiographic changes in response to canagliflozin treatment demonstrated favorable trends in positive cardiac remodeling, aligning with most available data in the literature.

## Conclusions

This study suggests that canagliflozin has a positive impact on hemodynamics and cardiac function. Specifically, it increases cardiac index, reduces systemic vascular resistance, improves diastolic function, and promotes favorable LA remodeling. These findings may help explain the beneficial effects of SGLT2 inhibitors on cardiovascular outcomes.

## Data Availability

The datasets used and analyzed during the current study are available from the corresponding author on reasonable request.

## Abbreviations

SGLT2i: Sodium-glucose co-transporter-2 inhibitors
T2DM: Type 2 diabetes
ICG: Impedance cardiography
GLS: Global longitudinal strain
HHF: Hospitalization for heart failure
HFrEF: Heart failure with reduced EF
HFpEF: Heart failure with preserved EF
